# The role of miR-10b-5p/brain-derived neurotrophic factor axis deregulation in poststroke epileptogenesis

**DOI:** 10.3389/fneur.2026.1735853

**Published:** 2026-02-18

**Authors:** Rainer Dormann, Joachim Gruber, Mariia Ragozina, David Demmel, Franziska Ammer-Pickhardt, Daniel Wallinger, Raimund Helbok, Tim J. von Oertzen, Anna R. Tröscher

**Affiliations:** 1 Department of Neurology, Kepler University Hospital, Johannes Kepler University Linz, Linz, Austria; 2 Clinical Research Institute for Neurosciences, Kepler University Hospital and Johannes Kepler University Linz, Linz, Austria; 3 Core Facility of Next Generation Sequencing, Johannes Kepler University Linz, Linz, Austria; 4 University Hospital Würzburg, Würzburg, Germany

**Keywords:** brain-derived neurotrophic factor (BDNF), ischemia, miRNome, post-stroke epilepsy, stroke recovery

## Abstract

**Introduction:**

Poststroke epilepsy (PSE) is a common complication following stroke and is associated with increased mortality and worse functional outcomes. There is no biomarker sufficiently to predict PSE, and antiseizure medications are initiated after the first unprovoked seizure. Early identification of patients at high risk for PSE is needed to consider preventive measures and improve management strategies.

**Methods:**

Illumina miRNA sequencing was performed on serum collected at follow-ups of patients with PSE and compared to ischemic stroke patients without epilepsy and patients with epilepsy without stroke (*N* = 24). Differentially expressed miRNAs were validated in a larger cohort (*N* = 53) by qPCR, and target prediction was performed *in silico*. Brain-derived neurotrophic factor (BDNF) levels were measured using ELISA and correlated with clinical parameters.

**Results:**

miRNA profiling revealed significant differences among the groups, with miR-10b-5p expression reduced in PSE patients compared to those with stroke alone. miR-486-5p was significantly reduced in PSE patients compared to epilepsy patients. qPCR validation confirmed miR-10b-5p as a potential biomarker candidate to distinguish PSE patients from stroke patients without PSE. BDNF, a key regulator of post-stroke recovery and epileptogenesis, was identified as a primary target of miR-10b-5p. While no group-level differences in serum BDNF concentrations were observed, BDNF levels correlated with disease duration and seizure latency exclusively in the PSE group.

**Discussion:**

Importantly, as samples were obtained during follow-up rather than the acute post-stroke phase, our results indicate an involvement of the miR-10b-5p/BDNF axis in long-term post-stroke remodeling or general PSE susceptibility rather than a predictive biomarker. However, the miR-10b-5p/BDNF axis may represent a biologically plausible pathway associated with post-stroke epileptogenesis and impaired post-ischemic recovery. Prospective longitudinal studies with early post-stroke sampling are required to determine its predictive value.

## Introduction

Post-stroke epilepsy (PSE) is a prevalent complication among stroke survivors, characterized by unprovoked seizures occurring beyond 7 days after the initial cerebrovascular event (1). The incidence of PSE varies, with studies indicating that approximately 10% of ischemic stroke patients develop epilepsy, and the majority of cases occur within the first 2 years following stroke (2). Several risk factors have been identified that increase the likelihood of developing PSE, such as the severity of stroke, involvement of cortical regions, previous early seizures, larger infarcts, or the presence of microbleeds (3, 4).

Early poststroke seizures occur within the first 7 days after stroke (5) and are thought to result from acute biochemical disruptions, such as increased extracellular potassium and glutamate concentrations, leading to neuronal hyperexcitability. In contrast, late seizures, which occur beyond this period, are associated with more permanent structural changes, including gliotic scarring, neurovascular unit dysfunction, and maladaptive plasticity, which contribute to sustained epileptogenesis (2).

The development of PSE has been associated with several adverse clinical outcomes. Patients with PSE have a higher mortality rate compared to those without seizures (6). Moreover, PSE is linked to poor functional recovery, increased post-stroke disability, and an elevated risk of dementia (7, 8).

Currently, there are no established guidelines for the primary prevention of PSE. The use of prophylactic antiseizure medications (ASMs) in the immediate post-stroke period is not routinely recommended due to the lack of evidence supporting their efficacy in preventing PSE and the potential for adverse effects such as cognitive decline or osteoporosis (9, 10). Management strategies focus on the prompt initiation of ASMs after the first unprovoked seizure (10).

To improve post-stroke outcomes, early stratification of patients prior to the occurrence of a first seizure would be highly beneficial. In this context, several studies have aimed to identify reliable biomarkers for predicting the development of PSE. Some have found genetic predispositions, such as polymorphisms in the CD40 (11), TRPM6 (12), or ALDH2 genes (13). Other studies reported correlations between protein levels in the blood and the likelihood of developing PSE, such as endostatin, Hsc70, S100B (14), TNFSF-14 (15), IL-1β (16), IL-6 (17), and neuropeptide Y (18). However, these biomarkers have yet to be validated, and many studies have focused on specific markers rather than using an unbiased approach.

To overcome this limitation, we used miRNA sequencing from blood samples to deliver a thorough analysis of the differences between ischemic stroke and PSE patients. miRNAs are small RNA molecules, typically 20–22 nucleotides in length, that can be isolated from various body fluids. Changes in the peripheral miRNome provide significant insights into pathophysiological alterations and possess potential for diagnostic applications (19–21). Considering the persistent challenge of identifying reliable markers for PSE, this study utilizes miRNA sequencing to enhance our understanding of PSE pathophysiology and identify potential targets for future biomarker research.

## Materials and methods

### Patients

Patients were retrospectively included between May 2021 and September 2023 from outpatient or inpatient presentations in accordance with the inclusion and exclusion criteria at the Department of Neurology, Kepler University Hospital, Linz. Four patient groups were predefined. The first group included patients who had been diagnosed with PSE. The second group included patients with ischemic stroke without the development of epileptic seizures within at least 2 years after stroke. The third group included patients with epilepsy without seizures in the last 2 weeks, and the fourth group was healthy controls. Patients aged between 18 and 99 years were included. Patients with severe comorbidity that can significantly influence the clinical presentation, such as known diseases from the spectrum of autoimmune diseases, patients with immunosuppressive medication, current drug abuse associated with epileptic seizures, previous structural gliotic cerebral lesions with cortical involvement visible on imaging other than the stroke lesions, vasculitis, previously known moderate to advanced dementia [Mini Mental State Examination (MMSE) ≤15 points] and malignant neoplasia were excluded. Furthermore, patients with pre-existing epilepsy before ischemia were excluded from the stroke and PSE group.

Patients were split into two cohorts. A total of 24 patients were used for miRNA-sequencing (epilepsy *n* = 9, stroke *n* = 7, PSE *n* = 8), and differentially expressed miRNAs were initially validated with these samples. To further validate our findings in a bigger cohort, we used the rest of the samples (epilepsy: *n* = 6, stroke: *n* = 8, PSE: *n* = 9, healthy controls: *n* = 5) to repeat the analysis of differentially expressed miRNAs and ELISA analysis. Details of which patient was allocated to which cohort can be found in Supplementary Table 1.

### Ethics

This study was approved by the Ethics Committee of the Johannes Kepler University, Linz, Austria (EK1066/2020; EK1151/2019, 1150/2019). All patients gave written informed consent.

### Clinical examination

Clinical data were collected during the clinical examination, from medical history, and from existing hospital documentation. The currently existing modified rating scale (mRS) is determined for patients in all groups on the basis of a clinical examination.

The age, gender, and mRS of all patients were recorded during check-ups. In the PSE group, as in the stroke group, the acute treatment of the ischemia, the mRS, the National Institutes of Health Stroke Scale (NIHSS) on admission and discharge, and the localization of the ischemia were recorded. In the PSE group, the duration from ischemia to the first epileptic seizure, as well as the seizure semiology and number of needed antiseizure medications, was recorded. In four patients, the interval could not be precisely determined, as the brain infarction occurred abroad (according to the patient, the seizure occurred within 1 year) or the patients were mute. The epilepsy diagnosis, with the localization of the suspected epileptogenic focus and the seizure semiology were recorded for epilepsy and PSE patients. The number of needed antiseizure medications was also included.

### Sample collection

For serum collection, blood was drawn from each patient and left at room temperature for 30 min before centrifugation at 1,000 × g for 15 min at 4 °C. Serum was then aliquoted in 500 μL and stored at −80 °C.

### Quality control of serum samples

To identify samples with a high level of hemolysis, we quantified the levels of hemoglobin in serum with NanodropOne by measuring absorbance at 414 nm. We used the custom plug-in provided by the company (Thermo Fisher; MA, United States) to directly calculate the concentration of hemoglobin. We excluded all samples with a calculated concentration of hemoglobin above 0.1 mg/mL, which is considered to be the cutoff for non-hemolyzed samples (Supplementary Figure 1A) (22).

### miRNA isolation

miRNA was isolated from 200 μL of serum with the miRNeasy Serum/Plasma Advanced kit from Qiagen (Hilden, Germany), following the manufacturer’s instructions, and eluted in 16 μL of water.

### Bioanalyzer

To evaluate the successful isolation of miRNAs from serum, we used the Bioanalyzer RNA 6000 Pico Kit (Agilent, CA, United States) according to the manufacturer’s instructions. A peak between 25 and 200 nt was assumed to correspond to small RNA species (Supplementary Figure 1B).

### qPCR hemolysis quality control

After miRNA isolation, we used qPCR for highly sensitive detection of hemolysis in the serum samples. To this end, we used 1.14 μL of eluate from the isolated RNA (equivalent to 16 μL of serum) and performed reverse transcription with the miRCURY LNA RT Kit from Qiagen (Hilden, Germany) according to the manufacturer’s instructions. For qPCR, cDNA was diluted 1:60, and the miRCURY LNA SYBR Green Kit (Qiagen, Hilden, Germany) was used with primers targeted against miR-23a-3p and miR-451a (miRNA qPCR Assay from Qiagen, Hilden, Germany). qPCR was performed on a BioRad CFX96 cycler under the following conditions: 95 °C for 2 min once, 95 °C for 10 s and 56 °C for 60 s, cycled 40 times in total. For the quantification of hemolysis, the difference between the two miRNAs was calculated. A difference below five is considered low hemolysis, between five and seven moderate, and above seven high hemolysis (23) (Supplementary Figure 1C).

### Library prep for small RNA sequencing

For library prep, the TruSeq small RNA Library Prep Kit from Illumina (CA, United States) was used. We used 5 μL of isolated miRNA as input for library prep and followed the manufacturer’s instructions. As the miRNA concentration in biofluids is usually low, we performed 15 PCR cycles for library amplification and added the optional library concentration step after gel purification. 2 nM library was used for sequencing.

### miRNA sequencing

All samples were sequenced together on an Illumina NextSeq 2000 device on a single NextSeq 1000/2000 P1 XLEAP-SBS Flowcell (100 Cycles) (# 20100983). The samples were loaded with a concentration of 650 pM and denatured on board. The following cycle settings were used: 6 cycles index read and 36 cycles insert read.

### miRNA sequencing analysis

The basecall was performed by the DRAGEN BCL Convert v4.2.7 pipeline of the DRAGEN server, locally integrated in the Illumina NextSeq 2000 device. The following analysis steps were then performed using CLC Genomics Workbench v24.0.2 (QIAGEN): trimming of reads by Phred score <13, trimming by ambiguous nucleotides >2, trimming by length <8, adapter trimming, quantification of miRNAs by aligning to miRBase v22 (*Homo sapiens*), and computation of differential expressions between the samples and sample groups with TMM normalization and FDR correction-filtering. This way, between 27,517 and 1,108,725 reads per sample could be associated with miRNAs described in miRNABase v22. The quality of sequencing results was evaluated with mirnaQC (24). A volcano plot of differentially expressed miRNAs (±1.5-fold change, *p*-value <0.05) was created with VolcanoseR for better graphical presentation of sequencing results but not used for selection of differentially expressed miRNAs (25). A Venn diagram was created with BioVenn (26). A heat map and hierarchical clustering of z-scores of selected miRNA expression were created with SRplot (27). For plotting single miRNA sequencing results, the read count of each miRNA of interest was normalized to the total read count of each sample.

### Validation of differentially expressed miRNAs by qPCR

To validate differentially expressed miRNAs by qPCR, we used a custom-designed miRCURY LNA PCR panel, which included two spike-in controls (UniSp6 and UniSp3) and SNORD68 (*Mus musculus*) as a negative control. As there is no clear consensus on reference genes for miRNAs in biofluids, we selected several potential reference miRNAs based on literature and sequencing data (miR-93-5p, miR-23a-3p, and U6snRNA) (28–31). miRNAs of interest were selected based on miRNA-seq data (miR-486-5p, miR-182-5p, miR-10b-5p, miR-10a-5p, 192-5p, miR-92-3p, miR-25-3p, miR-148-3p, miR-186-5p, and miR-145-5p). We used the previously generated cDNA and diluted it 1:40 according to the manufacturer’s protocol. miRCURY LNA SYBR Green Kit (Qiagen, Hilden, Germany) was used for qPCR. qPCRs were performed on a BioRad CFX96 cycler with the following conditions: 95 °C for 2 min once, followed by 40 cycles of 95 °C for 10 s and 56 °C for 60 s, followed by a standard melting curve analysis. All data were analyzed with Gene Globe (Qiagen, Hilden, Germany). We used the global mean to normalize Cq values of all samples.

### Target identification

We performed *in silico* analyses of miRNA targets. To this end, we investigated targets of miR-10b-5p based on three different tools: miRDB (32), TargetScan (33), and miRTargetLink 2.0 (34). We found that BDNF was one of the main targets of miR-10b-5p. In the next step, we investigated which of the differentially expressed miRNAs modulated BDNF expression in each group and manually counted them.

### BDNF ELISA

We performed an ELISA analysis of all serum samples for BDNF (BioLegend Cat. No 446604). Samples were diluted 1:10 or 1:20 for measurement. Absorbance was measured at 450 nm with a background correction at 570 nm with a standard plate reader (Tecan Sunrise). Frozen human brain tissue was used as a positive control, and cell culture medium from fibroblasts and keratinocytes as a negative control. Analysis was performed using a four-parameter logistic curve.

### Statistics

Statistical analysis was performed with GraphPad Prism (v.8). Analysis for the difference between the patient cohorts of clinical patient data was performed with one-way ANOVA for age and disease duration. For differences in scores (mRS and NIHSS), the Mann–Whitney *U*-test was performed. For the analysis of differential expression of normalized read counts, the Kruskal–Wallis test with Dunn’s correction for multiple testing was used. For qPCR results and ELISA, statistical testing was performed using one-way ANOVA with Holm–Šídák’s correction for multiple testing. To identify the required sample size (alpha value: 0.05, power: 0.95) for a validation cohort, we performed a power calculation between PSE and stroke based on the initial qPCR data and determined the required samples to be 13 per group. Correlation analyses were performed with Pearson’s correlation for all analyses except for the correlation between BDNF levels and mRS and NIHSS, where a Spearman’s correlation was used.

## Results

### Clinical examination

A total of 53 patients were included in the analysis, with a median age of 61.5 years (IQR 45.0–72.8), and 32% of the cohort were female. These patients were categorized into four groups: 16 with epilepsy, 15 with stroke without subsequent epilepsy, 17 with PSE, and 5 healthy controls. In the epilepsy group, the median age was 35.5 (IQR 24.17–59.03), in the stroke group, 73.0 (IQR 63.50–76.80), and in the PSE group, 61.55 (IQR 57.40–76.80), and in healthy controls, 51.24 (37.62–67.47, Table 1, more detailed information in Supplementary Table 1).

**Table 1:** Summary of patient data for all three groups.

	Epilepsy	Stroke	PSE	Healthy controls
Sex (m %)	11/16 (68%)	11/15 (73%)	12/17 (70%)	2/5 (40%)
Age (years; IQR)	35.35 (24.17–59.03)	73.00 (63.50–76.80)^###^	61.55 (57.40–73.25)^###^	51.25 (37.62–67.47)
Disease duration (years; IQR)	8.95 (2.42–10.95)	5.11 (2.44–13.19)	4.84 (0.79–6.55)	nA
NIHSS at first presentation (IQR)	nA	3.00 (2.00–10.00)	13.00 (7.50–17.50)	nA
NIHSS at discharge (IQR)	nA	1.00 (1.00–2.00)	9.00 (1.00–10.50)^**^	nA
mRS at first presentation (IQR)	nA	3.00 (1.00–5.00)	5.00 (3.00–5.00)	nA
mRS at discharge (IQR)	nA	1.00 (0.00–1.00)	4.00 (2.00–5.00)^**^	nA

Comparison of age and disease duration (one-way ANOVA, age: *F* = 13.39, *p* = 0.0001, PSE vs. stroke: *p* = 0.38, PSE vs. epilepsy: 0.0001, stroke vs. epilepsy: 0.001, epilepsy vs. HC: 0.23, PSE vs. healthy controls: 0.23, stroke vs. healthy controls: 0.09; disease duration: one-way ANOVA, *F* = 2.3, *p* = 0.11, PSE vs. stroke: *p* = 0.84, PSE vs. epilepsy: *p* = 0.11, stroke vs. epilepsy: *p* = 0.29). mRS and NIHSS are compared by the Mann–Whitney *U*-test (mRS at first presentation: *p* = 0.06, mRS at discharge: *p* = 0.044, NIHSS at first presentation: *p* = 0.0017, NIHSS at discharge: *p* = 0.0022). ^###^*p* < 0.001 comparison between epilepsy and stroke; ^**^p < 0.01 comparison stroke and PSE.

In the epilepsy group, 12 patients had focal epilepsy, of whom 8 also had secondary generalized seizures, and 4 patients had generalized seizures. One patient received triple antiseizure medication (ASM), 14 patients received monotherapy, and 1 patient no longer required ASM after years of seizure freedom (more details on seizure semiology and therapy in Supplementary Table 1).

The stroke group presented with a median mRS of 3.00 (IQR 1–5) and NIHSS of 3.0 (IQR 2.0–10). At discharge, functional outcomes had improved, with a median mRS of 1 (IQR 0.0–1.0) and a median NIHSS of 1 (IQR 0.0–2.0), reflecting largely favorable recovery (Table 1). Detailed information on lesion location and therapeutic measures for ischemia can be found in Supplementary Table 1.

The PSE group exhibited similar disability and neurological deficit scores both at admission and discharge. The mRS at admission was 5 (IQR 3–5), and the median NIHSS score was 13 (IQR 7–17), indicating moderate to severe neurological impairment. In contrast to the stroke group, the PSE group had a significantly worse functional status at discharge, with a median mRS of 4.0 (IQR 2.0–5.0), and the NIHSS decreased to 9 (IQR 1.0–11.0). The median latency between stroke and the first unprovoked seizure was 7.34 months (IQR 3.2–19.4). All patients had focal seizures, and three also experienced secondary generalized seizures. A total of 15 patients had a single ASM, 1 patient had dual therapy, and 1 was untreated after years of seizure freedom. Detailed information on lesion location, therapeutic measures for ischemia, and ASM can be found in Supplementary Table 1.

### Quality control of serum samples

We were able to isolate small RNA species from serum samples, as indicated by Bioanalyzer profiles showing a peak between 25 and 200 nt long RNA fragments (representative profile in Supplementary Figure 1B). As hemolysis can drastically alter the miRNA profile in serum samples, we measured the concentration of hemoglobin in all samples and used 0.1 mg/mL of hemoglobin as a cutoff (22). Our samples were all below the cutoff value (median epilepsy: 0.04, stroke: 0.03, PSE: 0.02; Supplementary Figure 1A). As a more sensitive measure for hemolysis, we also performed qPCR for miR-23a and miR-451a. Our samples were all below 7 (median epilepsy: 5.11, stroke: 4.79, PSE: 3.73; Supplementary Figure 1C) (23).

### miRNA sequencing quality control

Of the 24 samples sequenced, two did not pass the quality control. One sample had a raw read count of 7.14 *10^5^, and in one sample only 42% could be mapped to the reference genome (Supplementary Figures 1D,E). Excluding those two samples, the median number of raw reads per sample was 3.79 × 10^6^. The median number of detected miRNAs was 254.5, with an average length of 21.3 nt. A mean of 72.90% of the reads was identified as miRNAs, and 76.60% could be mapped to the reference genome. The average PhredScore was 29.6.

### miRNA sequencing reveals differences between epilepsy, stroke, and PSE

**Figure 1:**
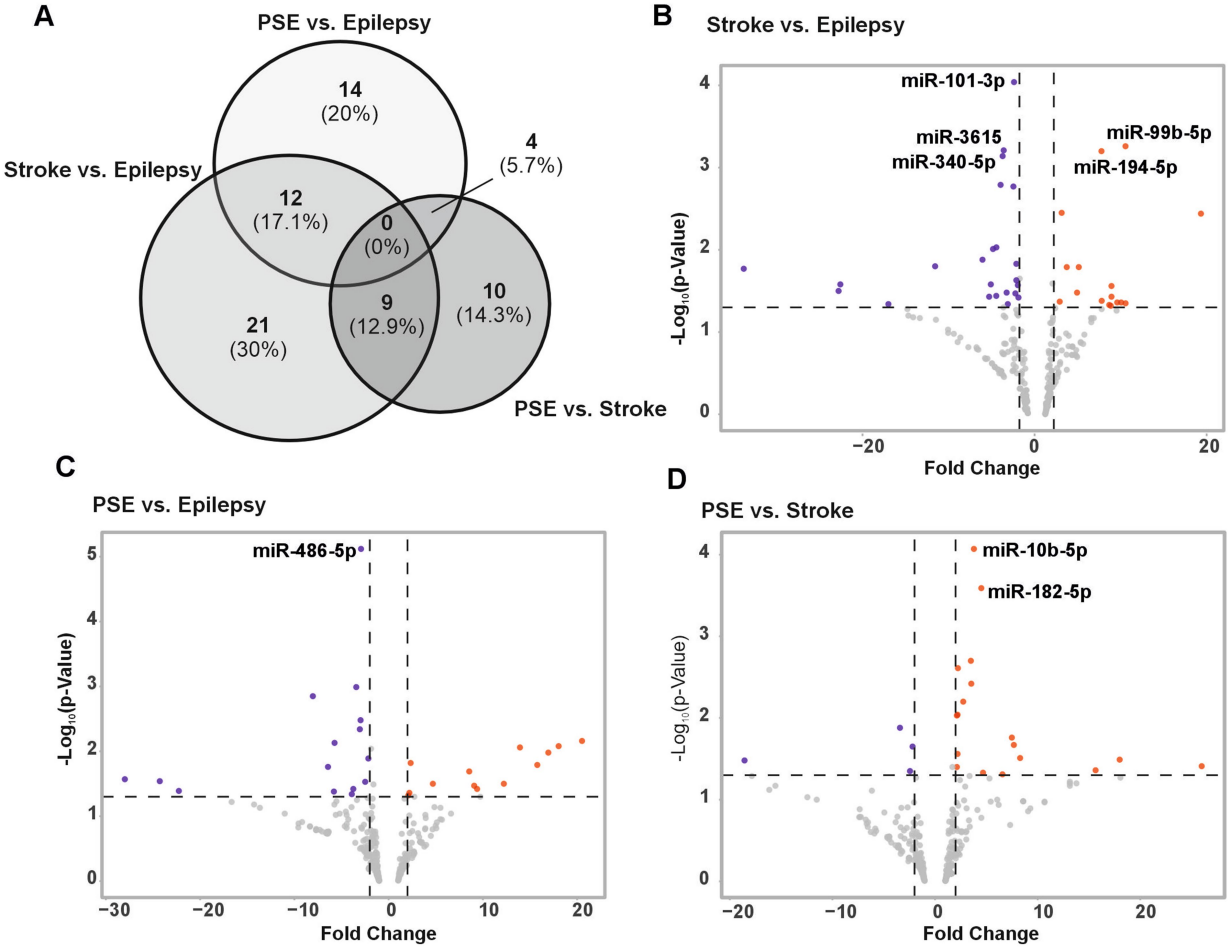
Overview of sequencing results. **(A)** Venn diagram of differentially expressed miRNAs (±1.5-fold change, *p* < 0.05) between PSE and epilepsy, PSE and stroke, and stroke and epilepsy. **(B)** Volcano plot of differentially expressed miRNAs (±1.5-fold change, *p* < 0.05) between stroke and epilepsy. miRNAs annotated in the graph were significantly altered with an FDR of <0.05. **(C)** Volcano plot of differentially expressed miRNAs (±1.5-fold change, *p* < 0.05) between PSE and epilepsy. miRNAs annotated in the graph were significantly altered with an FDR of <0.05. **(D)** Volcano plot of differentially expressed miRNAs (±1.5-fold change, *p* < 0.05) between PSE and stroke. miRNAs annotated in the graph were significantly altered with an FDR of <0.05.

**Figure 2:**
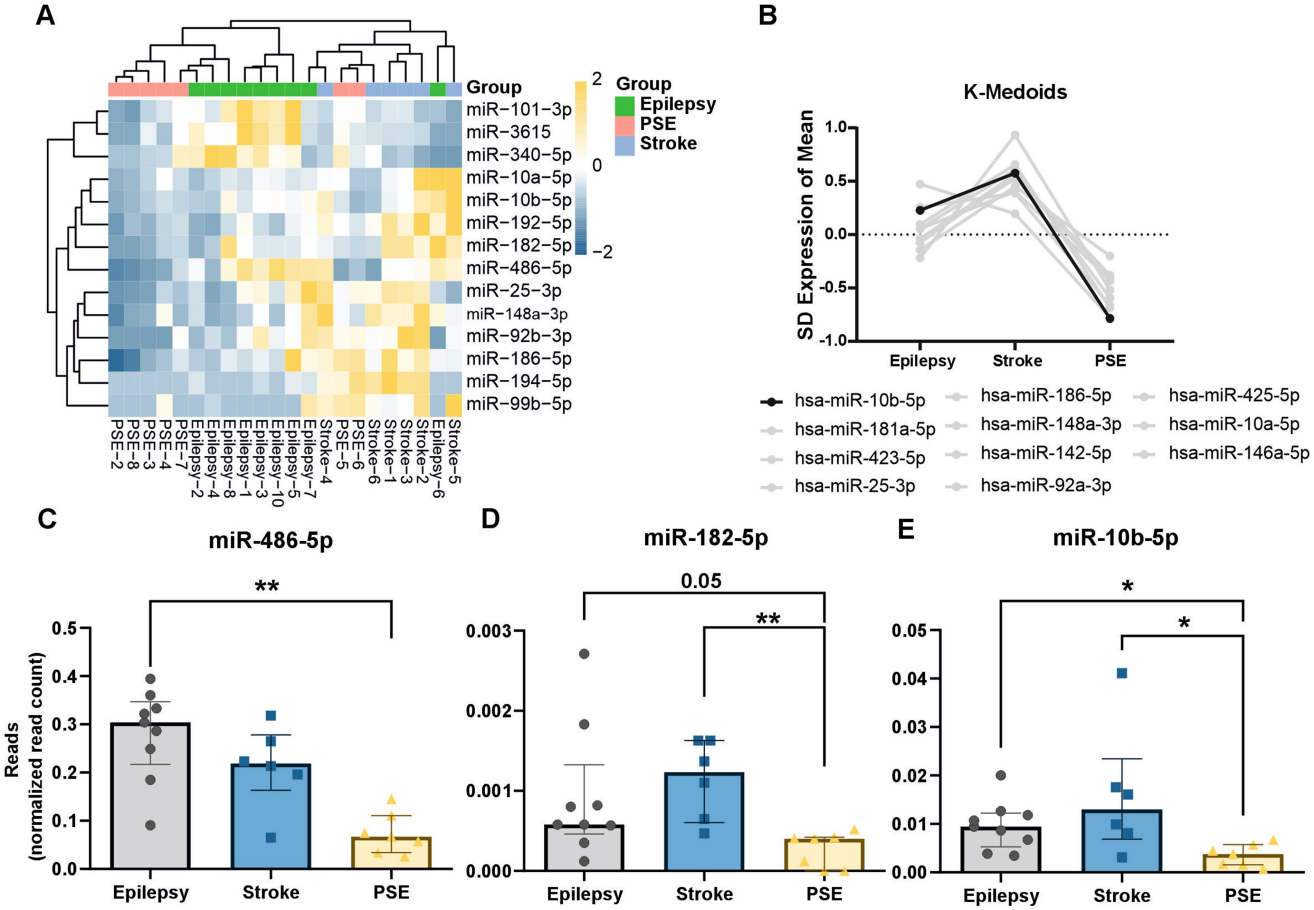
miR-10-5p is differentially expressed in PSE. **(A)** Hierarchical clustering of *z*-scores of selected miRNAs reveals similarities within the groups and a closer relation between epilepsy and PSE rather than PSE and stroke. **(B)** K-Medoids highlights several miRNAs differentiating between stroke and PSE. **(C)** miR-486-5p (normalized to overall read count) is significantly downregulated in PSE compared to epilepsy (Kruskal–Wallis test, Dunn’s *post-hoc* correction: *p*-value = 0.0005, Kruskal–Wallis statistics: 12.15; epilepsy vs. PSE *p*-value = 0.0015, stroke vs. PSE: *p* = 0.13, stroke vs. epilepsy: 0.7). **(D)** miR-182-5p is significantly downregulated in PSE compared to stroke and reaches near significance (*p* = 0.05) in the comparison between epilepsy and PSE (Kruskal–Wallis rest, Dunn’s *post-hoc* correction: *p*-value = 0.0031, Kruskal–Wallis statistics: 9.98; epilepsy vs. PSE *p*-value = 0.05, stroke vs. PSE: *p* = 0.0082, stroke vs. epilepsy: 0.99). **(E)** miR-10b-5p is significantly downregulated in PSE compared to stroke as well as to epilepsy (Kruskal–Wallis test, Dunn’s *post-hoc* correction: *p*-value = 0.012, Kruskal–Wallis statistics: 8.28; epilepsy vs. PSE *p*-value = 0.044, stroke vs. PSE: *p* = 0.02, stroke vs. epilepsy: 0.99). **(C–E)** Graphs are shown as median with interquartile range.

A total of 41 miRNAs were differentially expressed between stroke and epilepsy (Figures 1A,B), 30 miRNAs between epilepsy and PSE (Figures 1A,C), and 23 miRNAs between stroke and PSE (±2-fold change and a *p*-value of ≥0.05; Figures 1A,D). However, we only considered miRNAs with a ±2-fold change and an FDR value smaller than 0.05. When applying these stricter filtering criteria, only eight miRNAs were differently expressed among the three groups (annotated in Figures 1B–D). Between stroke and epilepsy, a total of five miRNAs were differentially expressed (miR-101-3p, miR-99-5p, miR-3651, miR-194-5p, and miR-340-5p; Figure 1B). In the comparison of PSE and epilepsy, only miR-486-5p was downregulated in PSE (Figure 1C). When comparing stroke with PSE, miR-10b-5p and miR-182-5p were upregulated in stroke (Figure 1D and Table 2; Supplementary Table 2). Based on the differentially expressed miRNAs, we performed hierarchical clustering of the samples and could show that most samples cluster well within each group, except for two PSE and one epilepsy case. Moreover, PSE samples cluster more closely to epilepsy samples rather than stroke samples, indicating a higher degree of similarity (Figure 2A). In a K-Medoids analysis, we found several miRNAs distinguishing the stroke and the PSE group, most prominently miR-10b-5p (Figure 2B). We then analyzed the miRNAs significantly changed in PSE individually. miR-486-5p was significantly upregulated in epilepsy compared to PSE (*p*-value = 0.0015), with no significant difference between stroke and PSE or epilepsy (*p*-value = 0.13 and 0.7; Figure 2C). miR-182-5p was significantly decreased in PSE compared to stroke (*p*-value = 0.008), with no significant difference between epilepsy and stroke (*p*-value >0.99) and a *p*-value of 0.05 between epilepsy and PSE (Figure 2D). miR-10b-5p was significantly increased in stroke and in epilepsy compared to PSE (epilepsy vs. PSE *p*-value = 0.04, stroke vs. PSE *p*-value = 0.02) with no significant difference between epilepsy and stroke (*p*-value >0.99; Figure 2E).

**Table 2:** Differentially expressed miRNAs.

Comparision	Name	Log2 fold change	Fold change	*p*-value	FDR *p*-value
PSE vs. epilepsy	hsa-miR-486-5p	−1.55	−2.93	7.59 × 10^−6^	8.2 × 10^−4^
Stroke vs. epilepsy	hsa-miR-101-3p	−1.39	−2.63	9.14 × 10^−5^	2.3 × 10^−2^
	hsa-miR-99b-5p	3.37	10.33	5.53 × 10^−4^	3.6 × 10^−2^
	hsa-miR-3615	−1.94	−3.83	6.20 × 10^−4^	3.6 × 10^−2^
	hsa-miR-194-5p	2.91	7.54	6.35 × 10^−4^	3.6 × 10^−2^
	hsa-miR-340-5p	−1.98	−3.95	7.17 × 10^−4^	3.6 × 10^−2^
Stroke vs. PSE	hsa-miR-10b-5p	1.92	3.79	8.61 × 10^−5^	7.1 × 10^−3^
	hsa-miR-182-5p	2.17	4.51	2.56 × 10^−4^	1.1 × 10^−2^

Summary of all miRNAs differentially expressed between the three groups (±2-fold change, FDR <0.05).

### miR-10b-5p distinguishes stroke and PSE

**Figure 3:**
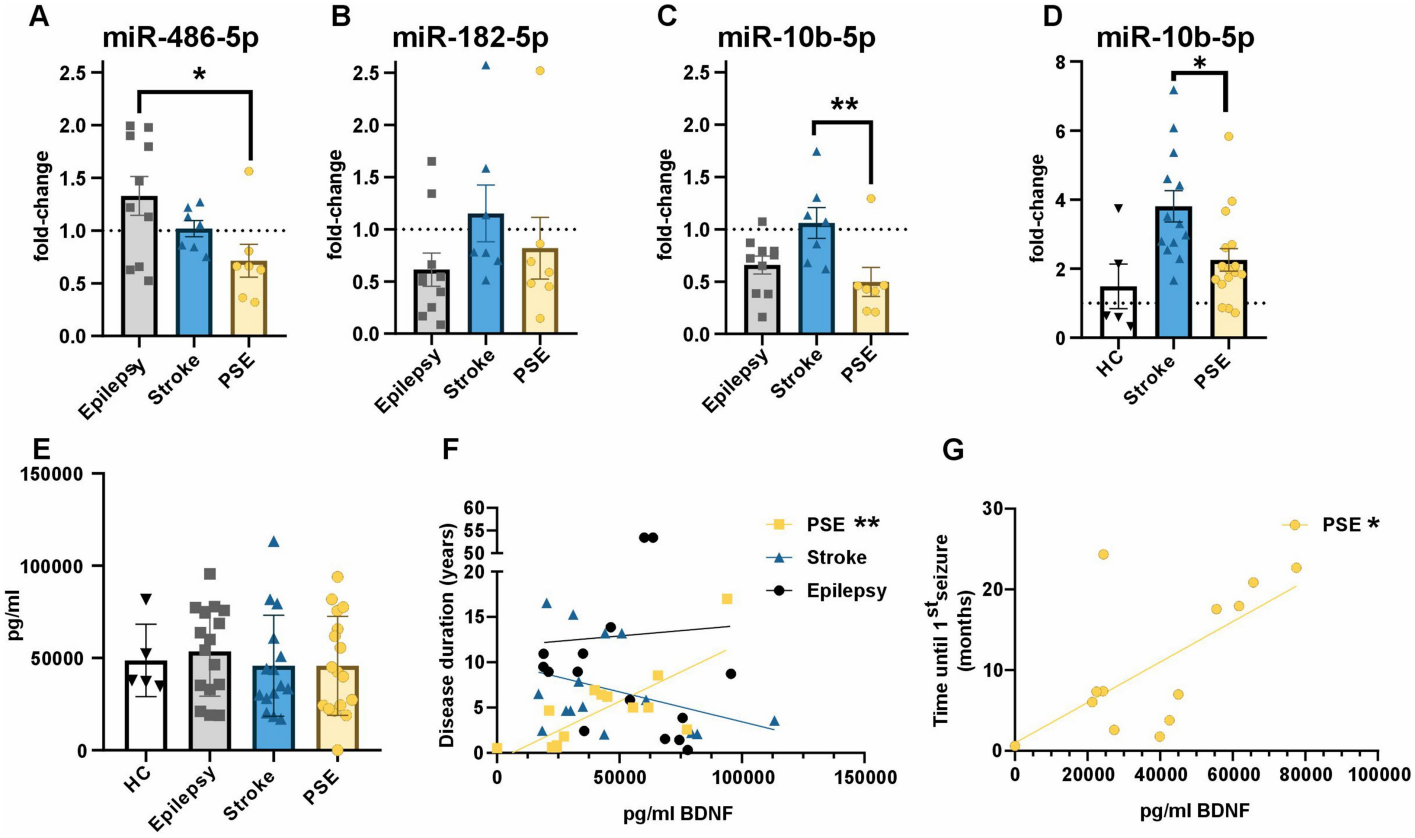
Validation of sequencing results by qPCR and ELISA. **(A)** miR-486-5p is significantly downregulated in PSE compared to epilepsy by qPCR (cohort 1, one-way ANOVA: *F* = 3.8, *p* = 0.04, PSE vs. epilepsy: *p* = 0.03, PSE vs. stroke: *p* = 0.44, epilepsy vs. stroke: 0.36). **(B)** miR-182-5p was not significantly altered between any of the groups by qPCR (cohort 1, one-way ANOVA: *F* = 1.4, *p* = 0.27, PSE vs. epilepsy: *p* = 0.8, PSE vs. stroke: *p* = 0.62, epilepsy vs. stroke: 0.24). **(C)** miR-10b-5p was significantly downregulated between PSE and stroke but not between PSE and epilepsy (cohort 1, one-way ANOVA: *F* = 5.22, *p* = 0.01, PSE vs. epilepsy: *p* = 0.6, PSE vs. stroke: *p* = 0.01, epilepsy vs. stroke: 0.06). **(D)** miR-10b-5p was significantly downregulated between PSE and stroke but not between PSE and healthy controls (cohorts 1 and 2, one-way ANOVA *F* = 6.23, *p* = 0.005, PSE vs. healthy controls: *p* = 0.53, PSE vs. stroke *p* = 0.015). **(E)** No difference in the concentration of BDNF in serum was found between any of the three groups (cohorts 1 and 2, one-way ANOVA: *F* = 0.32, *p* = 0.8, healthy control vs. epilepsy *p* = 0.98, healthy control vs. stroke *p* = 0.99, healthy control vs. PSE *p* = 0.99, epilepsy vs. stroke *p* = 0.83, epilepsy vs. PSE *p* = 0.82, stroke vs. PSE *p* = 0.99). **(F)** Disease duration was significantly correlated with BDNF concentration only in PSE (*R*^2^ = 0.56, *p* = 0.002), but not in epilepsy (*R*^2^ = 0.001, *p* = 0.9) or stroke (*R*^2^ = 0.13, *p* = 0.19). **(G)** Seizure latency (time between stroke and first seizure) was significantly correlated with BDNF concentration (*R*^2^ = 0.4, *p* = 0.019).

To validate the results from miRNA sequencing, we performed qPCR for differentially expressed miRNAs in cohort 1 (sequencing cohort). We found that miR-486-5p is significantly increased in epilepsy compared to PSE (*p*-value = 0.03), with no differences between PSE and stroke (*p*-value = 0.43) or epilepsy and stroke (*p*-value = 0.36), confirming results from sequencing (Figure 3A). We were not able to validate results for miR-182-5p and found no significant difference between any of the three groups (PSE vs. epilepsy: *p*-value = 0.8, PSE vs. stroke: *p*-value = 0.62, epilepsy vs. stroke: *p*-value = 0.24; Figure 3B). However, we could validate miR-10b-5p as a main candidate to differentiate stroke patients from PSE patients, as also by qPCR, miR-10b-5p was significantly decreased in PSE compared to stroke (*p*-value = 0.013). There was no significant difference between PSE and epilepsy (*p*-value = 0.6) or epilepsy and stroke (*p*-value = 0.06; Figure 3C). To rule out differences in miR-10b-5p expression due to the differences in age between the three groups, we calculated the correlation between the age and dCt values for miR-10b-5p and found no significant correlation (*R*^2^ = 0.01, *p* = 0.59, data are not shown).

As a next step, we pooled all patients from cohorts 1 and 2 to validate the differential expression of miR-10-p between stroke and PSE in a larger cohort by qPCR. We were able to show the differential expression of miR-10b-5p between PSE and stroke (*p*-value = 0.015), whereas we did not find a difference between PSE and healthy controls (*p*-value = 0.53, Figure 3D).

### BDNF as target of differentially expressed miRNAs

In the *in silico* analyses, we found that miR-10b-5p targets BDNF. Additionally, we found that of the 23 initially differentially expressed miRNAs between stroke and PSE, 6 (26%) bind to BDNF mRNA, namely miR-182-5p, miR-10a-5p, miR-192-5p, miR-16-5p, and let-7b-3p. In contrast, 4 of 30 (13%) of differentially expressed miRNAs were found to interact with BDNF in the comparison between PSE and epilepsy (miR-182-5p, miR-381-3p, miR-10b-5p, and miR-26b-3p), and only 3 of 41 (7%) in the comparison between stroke and epilepsy (miR-210-3p, miR-323a-3p, and miR-103a-3p; Supplementary Table 2).

### miR-10b-5p/BDNF axis involved in PSE development

We did not find a difference between any of the four groups in the ELISA analysis for BDNF (cohorts 1 and 2 pooled, Figure 3E, healthy control vs. epilepsy *p* = 0.98, healthy control vs. stroke *p* = 0.99, healthy control vs. PSE *p* = 0.99, PSE vs. epilepsy *p* = 0.82, PSE vs. stroke *p* = 0.99, epilepsy vs. stroke *p* = 0.83). However, we correlated BDNF levels with clinical parameters, namely age, NIHSS, mRS, disease duration, and time until the first seizure. We could show that in PSE, there was a significant direct correlation between BDNF levels and the disease duration (Figure 3F, *R*^2^ = 0.56, *p* = 0.002). However, this correlation was specific to PSE and could neither be found in epilepsy (*R*^2^ = 0.001, *p* = 0.9) nor in stroke (*R*^2^ = 0.13, *p* = 0.19). Furthermore, we found a correlation between BDNF levels and the time until the first seizure occurred after stroke (Figure 3G, *R*^2^ = 0.4, *p* = 0.02). Neither NIHSS nor mRS were correlated with BDNF levels, neither at admission nor at discharge for stroke (NIHSS admission: *R* = 0.26, *p* = 0.35; NIHSS discharge: *R* = 0.009, *p* = 0.97; mRS admission: *R* = 0.1, *p* = 0.72; mRS discharge: *R* = −0.2, *p* = 0.48) nor for PSE (NIHSS admission: *R* = −0.33, *p* = 0.19; NIHSS discharge: *R* = −0.26, *p* = 0.32; mRS admission: *R* = −0.35, *p* = 0.17; mRS discharge: *R* = −0.28, *p* = 0.28, Supplementary Figures 2B,C). We found no significant correlation between BDNF levels and age (Supplementary Figure 2A).

## Discussion

Poststroke epilepsy is a serious complication that necessitates vigilant monitoring and management. However, the underlying pathophysiologic mechanisms and prognostic biomarkers have not been well established (2). Given that miRNAs derived from liquid biopsies not only provide insight into pathophysiological processes within the CNS but also hold promise as diagnostic biomarkers (35, 36). We performed unbiased miRNA sequencing of serum collected at follow-ups from patients with stroke, epilepsy, and PSE to address these aspects systematically.

On average, we found 255 miRNAs per sample, of which we identified miR-10b-5p as a potential biomarker candidate to distinguish between patients with PSE and those with stroke without subsequent epileptic events. miR-10b-5p showed the most prominent alterations between PSE and stroke and was downregulated approximately 4-fold in PSE compared to stroke in the sequencing results and approximately 2-fold lower in the qPCR validation.

miR-10b-5p has previously been implicated in traumatic brain injury and has consistently been reported to be downregulated in epilepsy (37, 38). However, we did not find miR-10b-5p differentially expressed between epilepsy and stroke patients, suggesting that its alteration may be specific to poststroke conditions rather than epilepsy per se. Furthermore, miR-10b-5p has been shown to promote neuronal survival and regulate neuronal autophagy (39), providing a biologically plausible link to post-ischemic recovery processes. Hence, low levels of miR-10b-5p in PSE could point toward decreased neuronal survival in PSE patients compared to stroke patients. However, this also suggests that the severity of stroke itself could (also) have an influence on miR-10b-5p expression and should therefore be considered as a possible confounder. Importantly, stroke severity itself has consistently been identified as a major risk factor for the development of poststroke epilepsy in previous studies. Higher NIHSS scores, larger lesion size, cortical involvement, and poorer functional outcomes have all been associated with an increased risk of PSE, underscoring stroke severity as a clinically well-established determinant of epileptogenesis after ischemic injury (3, 52). Therefore, stroke severity represents a relevant potential confounder when interpreting the molecular alterations associated with PSE.

Nevertheless, given that miR-10b-5p levels did not differ between stroke patients without epilepsy and patients with epilepsy without stroke, a simple gradual decline due to disease severity alone appears unlikely. Instead, the observed expression pattern suggests that miR-10b-5p may reflect poststroke-specific maladaptive plasticity rather than epilepsy itself. How miR-10b-5p levels are affected directly after stroke and during recovery needs to be addressed in future studies.

The age difference between the group with PSE and the group with stroke without epilepsy may also influence miRNA expression. However, we did not find a correlation between miR-10b-5p levels and age, indicating that the observed differences are rather due to the underlying disease than differences in age. Moreover, this heterogeneity also corresponds to the wide range of stroke patients with varying degrees of severity and the wide age spectrum in which ischemia occurs, and thus reflects everyday clinical practice.

miRNAs exert their biological effects by regulating gene expression through interactions with target mRNAs. To gain further insights into possible pathophysiological processes differentiating PSE from stroke patients without epilepsy, we performed a target identification analysis of miR-10b-5p and found that one of its main targets is BDNF. BDNF is a neurotrophin and one of the most essential growth factors in the CNS. It plays a critical role in neuroplasticity, long-term potentiation, and neurodevelopment and has been implicated in several neurological disorders, such as neurodegenerative diseases, epilepsy, and multiple sclerosis (40, 41). BDNF expression is not only modified by miR-10b-5p but also by 26% of differentially expressed miRNAs found in this study in the comparison between stroke and PSE, namely miR-182-5p, miR-10a-5p, miR-192-5p, miR-16-5p, and let-7b-3p. Although we were only able to validate miR-10b-5p by qPCR in our small cohort, the involvement of the BDNF pathways warrants further investigation in larger, more powerful studies.

Given the high representation of BDNF-associated miRNAs, we proceeded to assess serum BDNF concentrations across all patient groups. Although no differences in BDNF levels were observed between the groups, a significant correlation between BDNF concentration and the disease duration within the PSE group was identified. In contrast, no such correlation was found in the epilepsy or stroke groups. In early PSE cases, we observed the lowest BDNF concentrations, which aligns with previous findings demonstrating reduced BDNF levels in the acute phase following stroke (42, 43). As BDNF levels did not differ significantly between stroke and PSE patients, neither in this study nor in others involving direct comparison (15). It is conceivable that the initial post-stroke reduction in BDNF is comparable in both groups, but that stroke patients without future development of PSE experience a faster recovery of BDNF levels over time. This is consistent with a previous finding indicating that a faster increase in BDNF concentration after stroke is linked with a better outcome (44). Furthermore, altered BDNF concentration has recently been associated with post-stroke depression (42, 45).

The effect of BDNF on epileptogenesis is highly discussed, as studies are showing a pro-epileptogenic effect in animal models and increased BDNF levels in the serum of epilepsy patients (41, 46, 47), whereas others report a neuroprotective, anti-epileptogenic effect and decreased BDNF levels in patients (48, 49). BDNF levels have been reported to decrease in the first 72 h after a tonic-clonic seizure but remain comparable to healthy controls in between seizures (49, 50).

We observed a positive correlation between BDNF concentration and the time between stroke and the first seizure. As BDNF quantification was performed on samples collected during follow-up rather than during the acute post-stroke phase, it is unlikely that the measured values directly reflect levels present during epileptogenesis. Instead, individual BDNF concentrations may influence post-stroke recovery trajectories and, in conjunction with lesion characteristics, contribute indirectly to the development of PSE.

It must be noted that the correlation between serum BDNF levels and its concentration in the CNS is incompletely understood. Peripheral BDNF may not adequately reflect CNS BDNF signaling, as peripheral sources, platelet release, and blood–brain barrier dynamics substantially influence circulating BDNF concentrations (51). Investigating BDNF concentrations in the CNS would be ideal. However, as lumbar puncture is not routinely performed in stroke patients, such investigations are only feasible in animal models and thus lack clinical relevance. Therefore, serum BDNF should be interpreted as an indirect marker of systemic or recovery-related processes rather than as a direct surrogate of cortical neurotrophic signaling.

Although current guidelines do not recommend prophylactic ASM use following stroke, biomarker-based stratification may open new avenues for individualized preventive strategies. Patients could in the future be identified as high-risk through miRNA profiling and be considered for targeted early interventions or prioritized for research in ASM prophylaxis trials. This paradigm would represent a shift toward precision neurology in stroke care.

A major strength of this study is the unbiased miRNA sequencing approach combined with targeted qPCR validation and extensive preanalytical quality control, including rigorous assessment of hemolysis. Furthermore, the inclusion of both stroke patients without epilepsy and epilepsy patients without stroke enabled a more specific interpretation of PSE-associated molecular alterations.

As this study only comprises a small patient cohort and all analyses were performed from samples collected at follow-ups, our findings regarding the role of the miR-10b-5p/BDNF axis should be interpreted as hypothesis-generating and therefore be viewed with caution and warrant validation in larger, ideally prospective, cohorts. Therefore, the identified alterations in miR-10b-5p should be interpreted as reflective of susceptibility or long-term post-stroke remodeling rather than as evidence for an early predictive biomarker.

Nevertheless, our cohort represented the clinical presentation of PSE patients well, as we found them to be more severely affected at discharge and at follow-ups compared to stroke patients without epilepsy, as indicated by significantly higher mRS and NIHSS scores at discharge (3). Importantly, no significant differences in clinical parameters were observed at admission between the stroke and PSE group. The more favorable improvement observed in the stroke cohort could indicate a less severe initial ischemic insult and, consequently, suggest that the miR-10b-5p/BDNF axis reflects a higher degree of stroke-related brain damage in the PSE group. However, given that stroke patients without epilepsy did not differ in miR-10b-5p levels from these from the epilepsy group, a gradual decline of miR-10b-5p expression solely driven by stroke severity appears unlikely.

Regarding seizure severity, patients with PSE and patients with epilepsy without stroke showed comparable clinical presentations. Both groups achieved effective seizure control with monotherapy in the majority of cases (75 and 80%, respectively), suggesting that seizure burden alone is unlikely to account for the observed molecular differences.

Despite clear limitations of this pilot study, including the small sample size, potential confounding by age and stroke severity, and sample collection during follow-up, we were able to identify the miR-10b-5p/BDNF axis as a possible biomarker candidate associated with PSE. Prospective longitudinal studies with early post-stroke sampling and repeated follow-up measurements are required to further elucidate the role of this pathway in post-ischemic epileptogenesis.

## Conclusion

In conclusion, unbiased miRNA sequencing enabled the identification of a novel, potential pathophysiological pathway contributing to the development of PSE. The miR-10b-5p/BDNF axis emerges as a potential biomarker candidate associated with increased susceptibility to PSE rather than as a validated predictive marker. Given the small sample size, the retrospective design with analyses performed after the development of poststroke epilepsy, and the heterogeneity of the included patients with respect to age, stroke severity, and seizure characteristics, these findings should be regarded as hypothesis-generating. Large prospective longitudinal studies with early post-stroke sampling and careful adjustment for relevant confounders are required to determine whether alterations in the miR-10b-5p/BDNF axis precede epileptogenesis or reflect long-term maladaptive post-stroke plasticity.

## Data Availability

The datasets presented in this study can be found in online repositories. The names of the repository/repositories and accession number(s) can be found in the article/Supplementary material.

## Supplementary material

SUPPLEMENTARY FIGURE 1 Quality control sequencing. **(A)** Hemoglobin concentration in epilepsy, stroke and PSE. **(B)** Representative graph of Bioanalyzer results from isolated miRNA from serum. **(C)** Analysis of hemolysis by qPCR for miR-23a and miR-451a with a cut-off indicated at 7. **(D)** Percentage of miRNA sequences derived from sequencing mapped to the reference genome. **(E)** Number of trimmed reads in all three groups.

SUPPLEMENTARY FIGURE 2 BDNF concentration was correlated with clinical parameter. **(A)** Age (Pearson correlation, PSE: *R*^2^ = 0.04, *p* = 0.47, epilepsy: *R*^2^ = 0.0, *p* = 0.85, stroke: *R*^2^ = −0.26, *p* = 0.34). **(B)** NIHSS (Spearman correlation, PSE presentation: *R* = −0.34, *p* = 0.19, stroke presentation: *R* = 0.26, *p* = 0.35, PSE discharge: *R* = −0.26, *p* = 0.32, stroke discharge: *R* = 0.01, *p* = 0.98). **(C)** mRS (Spearman correlation, PSE presentation: *R*^2^ = −0.35, *p* = 0.17, stroke presentation: *R* = 0.01, *p* = 0.73, PSE discharge: *R* = −0.27, *p* = 0.28, stroke discharge: *R* = −0.2, *p* = 0.48).

SUPPLEMENTARY TABLE 1 Detailed information on patients.

SUPPLEMENTARY TABLE 2 Detailed information on differentially expressed miRNAs.

## References

[1] Beghi E Carpio A Forsgren L Hesdorffer DC Malmgren K Sander JW . Recommendation for a definition of acute symptomatic seizure. Epilepsia. (2010) 51:671–5. doi: 10.1111/j.1528-1167.2009.02285.x, 19732133

[2] Tanaka T Ihara M Fukuma K Mishra NK Koepp MJ Guekht A . Pathophysiology, diagnosis, prognosis, and prevention of poststroke epilepsy: clinical and research implications. Neurology. (2024) 102:e209450. doi: 10.1212/WNL.0000000000209450, 38759128 PMC11175639

[3] Galovic M Döhler N Erdélyi-Canavese B Felbecker A Siebel P Conrad J . Prediction of late seizures after ischaemic stroke with a novel prognostic model (the SeLECT score): a multivariable prediction model development and validation study. Lancet Neurol. (2018) 17:143–52. doi: 10.1016/S1474-4422(17)30404-0, 29413315

[4] Gruber J Gattringer T Mayr G Schwarzenhofer D Kneihsl M Wagner J . Frequency and predictors of poststroke epilepsy after mechanical thrombectomy for large vessel occlusion stroke: results from a multicenter cohort study. J Neurol. (2023) 270:6064–70. doi: 10.1007/S00415-023-11966-X, 37658859 PMC10632247

[5] Scheffer IE Berkovic S Capovilla G Connolly MB French J Guilhoto L . ILAE classification of the epilepsies: position paper of the ILAE Commission for Classification and Terminology. Epilepsia. (2017) 58:512–21. doi: 10.1111/epi.13709, 28276062 PMC5386840

[6] Burneo JG Fang J Saposnik G. Impact of seizures on morbidity and mortality after stroke: a Canadian multi-centre cohort study. Eur J Neurol. (2010) 17:52–8. doi: 10.1111/J.1468-1331.2009.02739.X, 19686350

[7] Misra S Kasner SE Dawson J Tanaka T Zhao Y Zaveri HP . Outcomes in patients with poststroke seizures: a systematic review and meta-analysis. JAMA Neurol. (2023) 80:1155–65. doi: 10.1001/JAMANEUROL.2023.3240, 37721736 PMC10507596

[8] Samake B Houot M Zavanone C Vassilev K Thivard L Herlin B . Late but not early seizures impact negatively early post stroke recovery: a case-control study. Eur Stroke J. (2023) 8:784–91. doi: 10.1177/23969873231182493, 37329139 PMC10472947

[9] Holtkamp M Beghi E Benninger F Kälviäinen R Rocamora R Christensen H. European Stroke Organisation guidelines for the management of post-stroke seizures and epilepsy. Eur Stroke J. (2017) 2:103–15. doi: 10.1177/2396987317705536, 31008306 PMC6453212

[10] JosephsonCB WiebeS Delgado-GarciaG Gonzalez-IzquierdoA DenaxasS SajobiTT . Association of enzyme-inducing antiseizure drug use with long-term cardiovascular disease. JAMA Neurol. (2021) 78:1367–74. doi: 10.1001/JAMANEUROL.2021.3424, 34605857

[11] Zhang B Chen M Yang H Wu T Song C Guo R. Evidence for involvement of the CD40/CD40L system in post-stroke epilepsy. Neurosci Lett. (2014) 567:6–10. doi: 10.1016/j.neulet.2014.03.003, 24657679

[12] Fu C-Y Chen S-J Cai N-H Liu Z-H Zhang M Wang P-C . Increased risk of post-stroke epilepsy in Chinese patients with a TRPM6 polymorphism. Neurol Res. (2019) 41:378–83. doi: 10.1080/01616412.2019.1568755, 30739590

[13] Yang H Song Z Yang GP Zhang BK Chen M Wu T . The ALDH2 rs671 polymorphism affects post-stroke epilepsy susceptibility and plasma 4-HNE levels. PLoS One. (2014) 9:e109634. doi: 10.1371/journal.pone.0109634, 25313998 PMC4196934

[14] Abraira L Santamarina E Cazorla S Bustamante A Quintana M Toledo M . Blood biomarkers predictive of epilepsy after an acute stroke event. Epilepsia. (2020) 61:2244–53. doi: 10.1111/EPI.16648, 32857458

[15] Abraira L López-Maza S Quintana M Fonseca E Toledo M Campos-Fernández D . Exploratory study of blood biomarkers in patients with post-stroke epilepsy. Eur Stroke J. (2024) 9:763–71. doi: 10.1177/23969873241244584, 38557165 PMC11418466

[16] Zhang Q Li G Zhao D Yang P Shabier T Tuerxun T. Association between IL-1β and recurrence after the first epileptic seizure in ischemic stroke patients. Sci Rep. (2020) 10:13505. doi: 10.1038/s41598-020-70560-7, 32782321 PMC7419303

[17] Jia Q Jiang F Ma D Li J Wang F Wang Z. Association between IL-6 and seizure recurrence in patients with the first post-ischemic stroke seizure. Neuropsychiatr Dis Treat. (2020) 16:1955–63. doi: 10.2147/NDT.S257870, 32848401 PMC7429209

[18] Wang N Wang D Zhou H Xu C Hu X Qian Z . Serum neuropeptide Y level is associated with post-ischemic stroke epilepsy. J Stroke Cerebrovasc Dis. (2021) 30:105475. doi: 10.1016/J.JSTROKECEREBROVASDIS.2020.105475, 33242785

[19] Enright N Simonato M Henshall DC. Discovery and validation of blood microRNAs as molecular biomarkers of epilepsy: ways to close current knowledge gaps. Epilepsia Open. (2018) 3:427–36. doi: 10.1002/epi4.12275, 30525113 PMC6276772

[20] Maitrias P Metzinger-Le Meuth V Nader J Reix T Caus T Metzinger L. The involvement of miRNA in carotid-related stroke. Arterioscler Thromb Vasc Biol. (2017) 37:1608–17. doi: 10.1161/ATVBAHA.117.309233, 28775076

[21] Salvi V Gianello V Tiberio L Sozzani S Bosisio D. Cytokine targeting by miRNAs in autoimmune diseases. Front Immunol. (2019) 10:15. doi: 10.3389/fimmu.2019.00015, 30761124 PMC6361839

[22] Koseoglu M Hur A Atay A Çuhadar S. Effects of hemolysis interferences on routine biochemistry parameters. Biochem Med. (2011) 21:79–85. doi: 10.11613/bm.2011.015, 22141211

[23] Shah JS Soon PS Marsh DJ. Comparison of methodologies to detect low levels of hemolysis in serum for accurate assessment of serum microRNAs. PLoS One. (2016) 11:e0153200. doi: 10.1371/journal.pone.0153200, 27054342 PMC4824492

[24] Aparicio-Puerta E Gómez-Martín C Giannoukakos S Medina JM Marchal JA Hackenberg M. MirnaQC: a webserver for comparative quality control of miRNA-seq data. Nucleic Acids Res. (2020) 48:W262–7. doi: 10.1093/nar/gkaa452 32484556 PMC7319542

[25] Goedhart J Luijsterburg MS. VolcaNoseR is a web app for creating, exploring, labeling and sharing volcano plots. Sci Rep. (2020) 10:20560. doi: 10.1038/s41598-020-76603-3, 33239692 PMC7689420

[26] Hulsen T de Vlieg J Alkema W. BioVenn—a web application for the comparison and visualization of biological lists using area-proportional Venn diagrams. BMC Genomics. (2008) 9:1–6. doi: 10.1186/1471-2164-9-488 18925949 PMC2584113

[27] Tang D Chen M Huang X Zhang G Zeng L Zhang G . SRplot: a free online platform for data visualization and graphing. PLoS One. (2023) 18:e0294236. doi: 10.1371/JOURNAL.PONE.0294236, 37943830 PMC10635526

[28] Causin RL Pessoa-Pereira D Souza KCB Evangelista AF Reis RMV Fregnani JHTG . Identification and performance evaluation of housekeeping genes for microRNA expression normalization by reverse transcription-quantitative PCR using liquid-based cervical cytology samples. Oncol Lett. (2019) 18:4753–61. doi: 10.3892/OL.2019.10824, 31611985 PMC6781752

[29] Donati S Ciuffi S Brandi ML. Human circulating miRNAs real-time qRT-PCR-based analysis: an overview of endogenous reference genes used for data normalization. Int J Mol Sci. (2019) 20:4353. doi: 10.3390/IJMS20184353, 31491899 PMC6769746

[30] Korma W Mihret A Tarekegn A Chang Y Hwang D Tessema TS . Identification of circulating miR-22-3p and miR-93-5p as stable endogenous control in tuberculosis study. Diagnostics. (2020) 10:868. doi: 10.3390/DIAGNOSTICS10110868, 33114169 PMC7690830

[31] Niu Y Wu Y Huang J Li Q Kang K Qu J . Identification of reference genes for circulating microRNA analysis in colorectal cancer. Sci Rep. (2016) 6:35611. doi: 10.1038/SREP35611, 27759076 PMC5069661

[32] Chen Y Wang X. miRDB: an online database for prediction of functional microRNA targets. Nucleic Acids Res. (2020) 48:D127–31. doi: 10.1093/NAR/GKZ757 31504780 PMC6943051

[33] McGeary SE Lin KS Shi CY Pham TM Bisaria N Kelley GM . The biochemical basis of microRNA targeting efficacy. Science. (2019) 366:eaav1741. doi: 10.1126/SCIENCE.AAV1741, 31806698 PMC7051167

[34] Kern F Aparicio-Puerta E Li Y Fehlmann T Kehl T Wagner V . miRTargetLink 2.0—interactive miRNA target gene and target pathway networks. Nucleic Acids Res. (2021) 49:W409–16. doi: 10.1093/NAR/GKAB297, 34009375 PMC8262750

[35] Szydlowska K Bot A Nizinska K Olszewski M Lukasiuk K. Circulating microRNAs from plasma as preclinical biomarkers of epileptogenesis and epilepsy. Sci Rep. (2024) 14:708. doi: 10.1038/s41598-024-51357-4, 38184716 PMC10771472

[36] Wang P Huang J Wen H Liang X Wang J Ding P . Clinical significance of GABA, NSE, and miR-155 expression in patients with post-stroke epilepsy. Neuroscience. (2025) 571:151–8. doi: 10.1016/J.NEUROSCIENCE.2025.01.057, 39890053

[37] Hu K Zhang C Long L Long X Feng L Li Y . Expression profile of microRNAs in rat hippocampus following lithium–pilocarpine-induced status epilepticus. Neurosci Lett. (2011) 488:252–7. doi: 10.1016/J.NEULET.2010.11.040, 21094214

[38] Kaalund SS Venø MT Bak M Møller RS Laursen H Madsen F . Aberrant expression of miR-218 and miR-204 in human mesial temporal lobe epilepsy and hippocampal sclerosis—convergence on axonal guidance. Epilepsia. (2014) 55:2017–27. doi: 10.1111/EPI.12839, 25410734

[39] Liu S Liu H Gong C Li G Li Q Pan Z . MiR-10b-5p regulates neuronal autophagy and apoptosis induced by spinal cord injury through UBR7. Neuroscience. (2024) 543:13–27. doi: 10.1016/J.NEUROSCIENCE.2024.02.013, 38382692

[40] Nagahara AH Tuszynski MH. Potential therapeutic uses of BDNF in neurological and psychiatric disorders. Nat Rev Drug Discov. (2011) 10:209–19. doi: 10.1038/nrd3366, 21358740

[41] Saad HM El-Saber Batiha G Alruwaili R Al-Kuraishy HM Al-Gareeb AI Ali NH . The possible role of brain-derived neurotrophic factor in epilepsy. Neurochem Res. (2023) 49:533–47. doi: 10.1007/S11064-023-04064-X, 38006577 PMC10884085

[42] Mojtabavi H Shaka Z Momtazmanesh S Ajdari A Rezaei N. Circulating brain-derived neurotrophic factor as a potential biomarker in stroke: a systematic review and meta-analysis. J Transl Med. (2022) 20:126. doi: 10.1186/s12967-022-03312-y 35287688 PMC8919648

[43] Øverberg LT Lugg EF Gaarder M Langhammer B Thommessen B Rønning OM . Plasma levels of BDNF and EGF are reduced in acute stroke patients. Heliyon. (2022) 8:e09661. doi: 10.1016/J.HELIYON.2022.E09661, 35756121 PMC9218156

[44] Li Y Han X Luo S Huang H Huang X Li M . Predictive value of longitudinal changes of serum matrix metalloproteinase-9 and brain-derived neurotrophic factor in acute ischemic stroke. Front Aging Neurosci. (2022) 14:952038. doi: 10.3389/FNAGI.2022.952038 36092813 PMC9452807

[45] Chang X He Y Liu Y Fei J Qin X Song B . Serum brain derived neurotrophic factor levels and post-stroke depression in ischemic stroke patients. J Affect Disord. (2024) 361:341–7. doi: 10.1016/J.JAD.2024.06.050, 38897298

[46] Alvim MKM Morita-Sherman ME Yasuda CL Rocha NP Vieira ÉL Pimentel-Silva LR . Inflammatory and neurotrophic factor plasma levels are related to epilepsy independently of etiology. Epilepsia. (2021) 62:2385–94. doi: 10.1111/EPI.17023, 34331458

[47] Demir M Akarsu EO Dede HO Bebek N Yıldız SO Baykan B . Investigation of the roles of new antiepileptic drugs and serum BDNF levels in efficacy and safety monitoring and quality of life: a clinical research. Curr Clin Pharmacol. (2020) 15:49–63. doi: 10.2174/1574884714666190312145409, 30864528 PMC7497568

[48] Chen NC Chuang YC Huang CW Lui CC Lee CC Hsu SW . Interictal serum brain-derived neurotrophic factor level reflects white matter integrity, epilepsy severity, and cognitive dysfunction in chronic temporal lobe epilepsy. Epilepsy Behav. (2016) 59:147–54. doi: 10.1016/J.YEBEH.2016.02.029, 27152461

[49] Poniatowski ŁA Cudna A Kurczych K Bronisz E Kurkowska-Jastrzębska I. Kinetics of serum brain-derived neurotrophic factor (BDNF) concentration levels in epileptic patients after generalized tonic-clonic seizures. Epilepsy Res. (2021) 173:106612. doi: 10.1016/J.EPLEPSYRES.2021.106612, 33774427

[50] Hong Z Li W Qu B Zou X Chen J Sander JW . Serum brain-derived neurotrophic factor levels in epilepsy. Eur J Neurol. (2014) 21:57–64. doi: 10.1111/ENE.12232 23879572

[51] Zheng Z Zhang L Zhu T Huang J Qu Y Mu D. Peripheral brain-derived neurotrophic factor in autism spectrum disorder: a systematic review and meta-analysis. Sci Rep. (2016) 6:31241. doi: 10.1038/srep31241, 27506602 PMC4979025

[52] Hesdorffer DC Benn EK Cascino GD Hauser WA. Is a first acute symptomatic seizure epilepsy? Mortality and risk for recurrent seizure. Epilepsia. (2009) 50:1102–8. doi: 10.1111/j.1528-1167.2008.01945.x, 19374657

