# Supplementary figures and images for: The role of miR-10b-5p/brain-derived neurotrophic factor axis deregulation in poststroke epileptogenesis
Source: Front Neurol. 2026 Feb 18;17:1735853. doi: 10.3389/fneur.2026.1735853 (PMC12956722; doi:10.3389/fneur.2026.1735853)

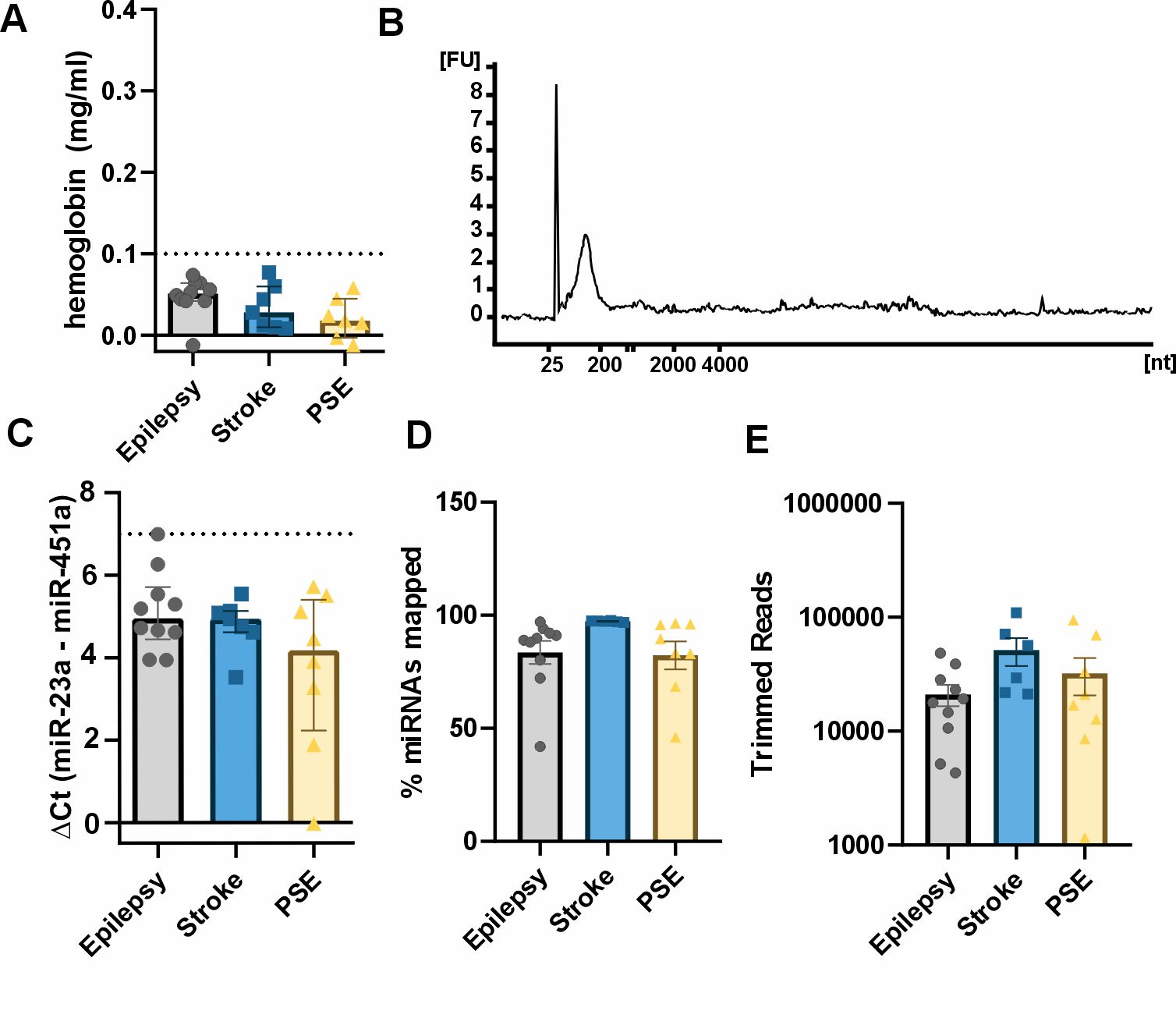

Supplement: Supplementary file 1 [file Image_1.jpeg]

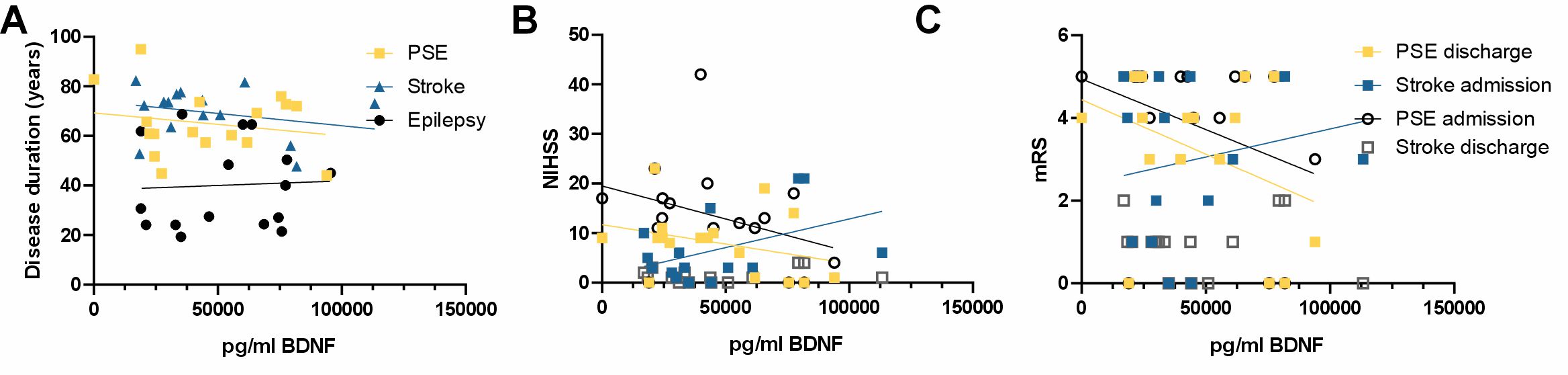

Supplement: Supplementary file 2 [file Image_2.jpeg]
